# Correction: Tracking the incidence and risk factors for SARS-CoV-2 infection using historical maternal booking serum samples

**DOI:** 10.1371/journal.pone.0308512

**Published:** 2024-08-01

**Authors:** Edward Mullins, Ruth McCabe, Sheila M. Bird, Paul Randell, Marcus J. Pond, Lesley Regan, Eleanor Parker, Myra McClure, Christl A. Donnelly

In S1 File, the Supplementary Figure 3 should have not been a duplicate of Supplementary Figure 2. Please view the correct S1 File below.

## Supporting information

S1 File (PDF)
