# Supplementary material for: Tracking the incidence and risk factors for SARS-CoV-2 infection using historical maternal booking serum samples
Source: PLoS One. 2022 Sep 2;17(9):e0273966. doi: 10.1371/journal.pone.0273966 (PMC9439206; doi:10.1371/journal.pone.0273966)
Supplement: S1 File — (DOCX) [file pone.0273966.s001.docx]

**Tracking the incidence and risk factors for SARS-CoV-2 infection using historical maternal booking serum samples**

**Supplementary Material**

Edward W. S. Mullins*^¶^, Ruth McCabe^¶^, Sheila M. Bird, Paul Randell, Marcus J. Pond, Lesley Regan, Eleanor Parker, Myra McClure, Christl A. Donnelly

^¶^ These authors contributed equally to this work.

*Corresponding author

Email: [edward.mullins@imperial.ac.uk](mailto:edward.mullins@imperial.ac.uk).

**Sample size**

Supplementary Table 1 presents an overview of the number of samples categorised as ‘clearly’ and ‘borderline’ seroreactive or non-seroreactive.

**Supplementary Table 1: Classification and sample sizes of observations from October 2019 to September 2020 as seroreactive and non-seroreactive according to different thresholds of binding ratio (BR) values.**

| **Assay threshold binding ratios (BRs)** | **Interpretation** | **Threshold sample size** | **Cumulative sample size** |
| --- | --- | --- | --- |
| BR ≥ 1.2 | Clearly seroreactive | 665 | 665 |
| 1.2 > BR ≥ 1 | Borderline seroreactive | 35 | 700 |
| 1 > BR ≥ 0.8 | Borderline non-seroreactive | 70 | 770 |
| 0.8 > BR | Clearly non-seroreactive | 10,486 | 11,256 |

**Estimating prevalence of seroreactivity**

Let $n$ be the total number of observations. For each observation $i$, $Y_{i}$ denotes whether this observation is seroreactive or non-seroreactive:

$$Y_{i}=\left\{ \begin{aligned} 1 if observation is seroreactive \\ 0 if observation is non-seroreactive \end{aligned} \right.$$

Each observation can be considered an independent Bernoulli trial for seroreactivity with probability $s$, where $s$ denotes prevalence of seroreactivitiy. It follows that the total number of seroreactive observations, $X=x$, is a binomially-distributed random variable with parameters $n$ and $s$:

$$X=\sum_{i=1}^{n} Y_{i}\sim Binomial(n,s)$$

Therefore, prevalence of seroreactivity was naively estimated using the maximum likelihood estimator (MLE) of the binomial distribution as follows:

$$\hat{s}=\frac{x}{n}$$

where the number of reactive observations was measured according to clearly and borderline seroreactive observations (binding ratio (BR) value ≥ 1). 95% confidence intervals were generated using the exact binomial method by solving the following equations for $s_{L}$ and $s_{U}$, respectively:

$$\sum_{k=0}^{x} \binom{n}{k}s_{U}^{k}\left( 1-s_{U} \right)^{n-k}=\frac{\alpha}{2}$$

$$\sum_{k=0}^{x-1} \binom{n}{k}s_{L}^{k}\left( 1-s_{L} \right)^{n-k}=1-\frac{\alpha}{2}$$

where $\alpha=0.05$.

**Fisher’s exact test for prevalence in June 2019 compared to main study period**

Supplementary Table 2 presents the results of the two-sided Fisher’s exact tests to test for the significance in the odds of a seroreactive sample between June 2019 compared to each fortnight in the main study.

**Supplementary Table 2: Results of two-sided Fisher’s exact test in the odds of observing a seroreactive sample in June 2019 (1000 samples, 8 positives) in comparison to each fortnight in the main study period (October 2019 – September 2020).**

| **Collection fortnight (fortnight-year)** | **Calendar date** | **Total observations (**$\boldsymbol{n}$**)** | **Seroreactive observations (**$\boldsymbol{x}$**)** | **Odds Ratio (MLE (95% exact confidence intervals))** | **p-value** |
| --- | --- | --- | --- | --- | --- |
| 22-2019 | 22/10/2019 - 04/11/2019 | 608 | 8 | 0.61 (0.20 – 1.86) | 0.313 |
| 23-2019 | 05/11/2019 - 18/11/2019 | 610 | 5 | 0.98 (0.28 – 3.81) | 1.000 |
| 24-2019 | 19/11/2019 - 02/12/2019 | 546 | 6 | 0.73 (0.22 – 2.55) | 0.581 |
| 25-2019 | 03/12/2019 - 16/12/2019 | 514 | 3 | 1.38 (0.33 – 8.07) | 0.759 |
| 26-2019 | 17/12/2019 - 31/12/2019 | 554 | 5 | 0.89 (0.25 – 3.46) | 0.781 |
| 1-2020 | 01/01/2020 - 14/01/2020 | 467 | 5 | 0.75 (0.21 – 2.91) | 0.565 |
| 2-2020 | 15/01/2020 - 28/01/2020 | 572 | 6 | 0.76 (0.23 – 2.67) | 0.590 |
| 3-2020 | 29/01/2020 - 11/02/2020 | 669 | 7 | 0.76 (0.24 – 2.48) | 0.607 |
| 4-2020 | 12/02/2020 - 25/02/2020 | 530 | 9 | 1.27 (0.47 – 0.16) | 0.127 |
| 5-2020 | 26/02/2020 - 10/03/2020 | 628 | 7 | 0.72 (0.23 – 2.33) | 0.597 |
| 6-2020 | 11/03/2020 - 24/03/2020 | 659 | 18 | 0.29 (0.11 – 0.70) | 0.004 |
| 7-2020 | 25/03/2020 - 07/04/2020 | 392 | 34 | 0.09 (0.03 – 0.19) | <0.001 |
| 8-2020 | 08/04/2020 - 21/04/2020 | 622 | 73 | 0.06 (0.03 – 0.13) | <0.001 |
| 9-2020 | 22/04/2020 - 05/05/2020 | 637 | 77 | 0.06 (0.02 – 0.12) | <0.001 |
| 10-2020 | 06/05/2020 - 19/05/2020 | 584 | 83 | 0.05 (0.02 – 0.10) | <0.001 |
| 11-2020 | 20/05/2020 - 02/06/2020 | 482 | 53 | 0.07 (0.03 – 0.14) | <0.001 |
| 12-2020 | 03/06/2020 - 16/06/2020 | 499 | 58 | 0.06 (0.03 – 0.13) | <0.001 |
| 13-2020 | 17/06/2020 - 30/06/2020 | 278 | 38 | 0.05 (0.02 – 0.11) | <0.001 |
| 14-2020 | 01/07/2020 - 14/07/2020 | 254 | 37 | 0.05 (0.02 – 0.11) | <0.001 |
| 15-2020 | 15/07/2020 - 28/07/2020 | 288 | 35 | 0.06 (0.02 – 0.13) | <0.001 |
| 16-2020 | 29/07/2020 - 11/08/2020 | 267 | 40 | 0.05 (0.02 – 0.10) | <0.001 |
| 17-2020 | 12/08/2020 - 25/08/2020 | 198 | 25 | 0.06 (0.02 – 0.13) | <0.001 |
| 18-2020 | 26/08/2020 - 08/09/2020 | 233 | 35 | 0.05 (0.02 – 0.10) | <0.001 |
| 19-2020 | 09/09/2020 - 22/09/2020 | 165 | 33 | 0.03 (0.01 – 0.07) | <0.001 |

**Logistic regression**

The logistic regression model was formally described as:

$$logit\left( s \right)=\beta_{0} +\beta_{1}Age_{18-29}+\beta_{2}Age_{30-34}$$

$$+\beta_{3}Ethnicity_{all-black}+\beta_{4}Ethnicity_{all-Asian}+\beta_{5}Ethnicity_{other}$$

$$+ \beta_{6}IMD_{deciles 3-6}+\beta_{7}IMD_{deciles 7-10}$$

$$+\beta_{8}Fortnight_{2019-22}+\beta_{9}Fortnight_{2019-23}+\beta_{10}Fortnight_{2019-24}+\beta_{11}Fortnight_{2019-25}+\beta_{12}Fortnight_{2019-26}+\beta_{13}Fortnight_{2020-1}+\beta_{14}Fortnight_{2020-2}+\beta_{15}Fortnight_{2020-3}+\beta_{16}Fortnight_{2020-4}+\beta_{17}Fortnight_{2020-5}+\beta_{18}Fortnight_{2020-7}+\beta_{19}Fortnight_{2020-8}+\beta_{20}Fortnight_{2020-9}+\beta_{21}Fortnight_{2020-10}+\beta_{22}Fortnight_{2020-11}+\beta_{23}Fortnight_{2020-12}+\beta_{24}Fortnight_{2020-13}+\beta_{25}Fortnight_{2020-14}+\beta_{26}Fortnight_{2020-15}+\beta_{27}Fortnight_{2020-16}+\beta_{28}Fortnight_{2020-17}+\beta_{29}Fortnight_{2020-18}+\beta_{30}Fortnight_{2020-19}$$

The addition model with the interaction term between ethnicity and IMD decile was as follows:

$$logit\left( s \right)=\beta_{0} +\beta_{1}Age_{18-29}+\beta_{2}Age_{30-34}$$

$$+\beta_{3}Ethnicity_{all-black}+\beta_{4}Ethnicity_{all-Asian}+\beta_{5}Ethnicity_{other}$$

$$+ \beta_{6}IMD_{deciles 3-6}+\beta_{7}IMD_{deciles 7-10}$$

$$+\beta_{8}Fortnight_{2019-22}+\beta_{9}Fortnight_{2019-23}+\beta_{10}Fortnight_{2019-24}+\beta_{11}Fortnight_{2019-25}+\beta_{12}Fortnight_{2019-26}+\beta_{13}Fortnight_{2020-1}+\beta_{14}Fortnight_{2020-2}+\beta_{15}Fortnight_{2020-3}+\beta_{16}Fortnight_{2020-4}+\beta_{17}Fortnight_{2020-5}+\beta_{18}Fortnight_{2020-7}+\beta_{19}Fortnight_{2020-8}+\beta_{20}Fortnight_{2020-9}+\beta_{21}Fortnight_{2020-10}+\beta_{22}Fortnight_{2020-11}+\beta_{23}Fortnight_{2020-12}+\beta_{24}Fortnight_{2020-13}+\beta_{25}Fortnight_{2020-14}+\beta_{26}Fortnight_{2020-15}+\beta_{27}Fortnight_{2020-16}+\beta_{28}Fortnight_{2020-17}+\beta_{29}Fortnight_{2020-18}+\beta_{30}Fortnight_{2020-19}+\beta_{31}Ethnicity_{all-black}\times{IMD}_{deciles 3-6}+\beta_{32}Ethnicity_{all-Asian}\times{IMD}_{deciles 3-6} +\beta_{33}Ethnicity_{other}\times{IMD}_{deciles 3-6} +\beta_{34}Ethnicity_{all-black}\times{IMD}_{deciles 7-10}+\beta_{35}Ethnicity_{all-Asian}\times{IMD}_{deciles 7-10} +\beta_{36}Ethnicity_{other}\times{IMD}_{deciles 7 -10}$$

**Baseline fortnight selection**

Fortnight 6 was just before the first UK lockdown in March 2020, was likely to coincide with high rates of infection and was selected as the baseline fortnight.

**Incidence of seroreactivity**

Supplementary Figure 1 presents the shape constrained P-spline fitted to the estimates of prevalence of seroreactivity over time, in which monotonicity between knots was enforced. In this instance, we specified 24 knots, corresponding to the number of fortnights for which incidence was estimated. The smoothed curve provided the estimates from which incidence was derived.

**Supplementary Figure 1: Shape constrained P-spline (red) fit to prevalence of seroreactivity estimated using entire cleaned data set (black).**


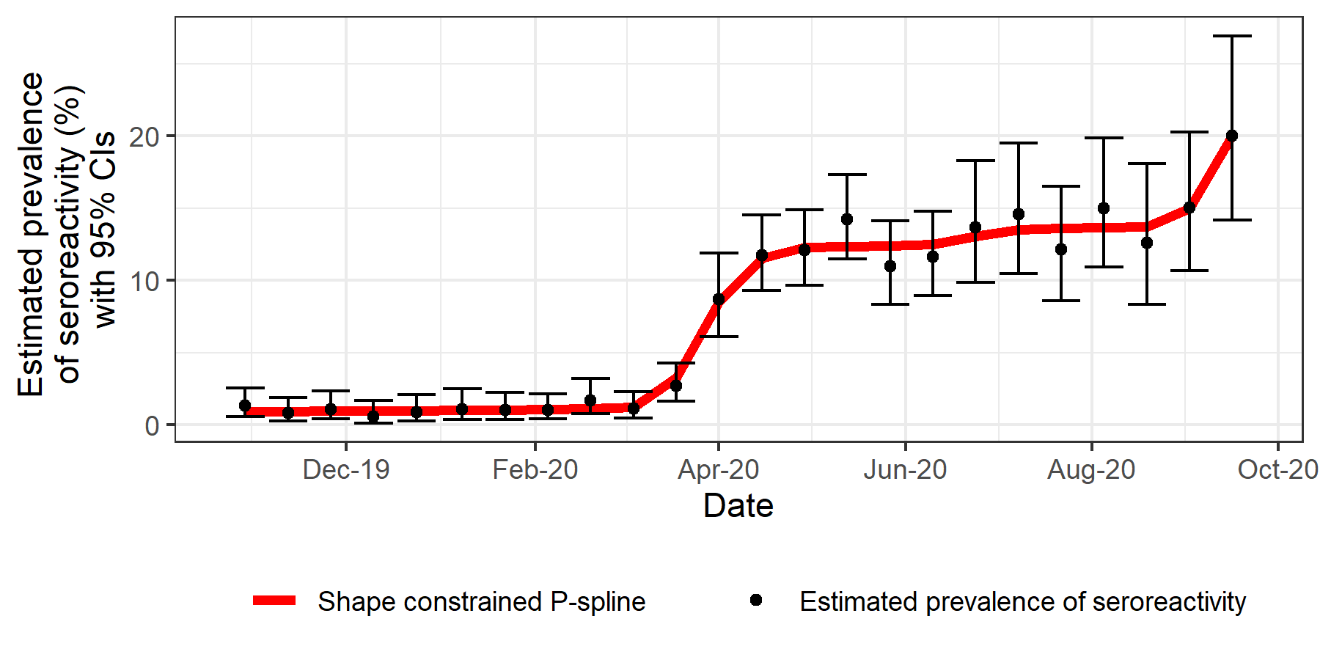


The bootstrapping procedure is presented below.

Let $f=1,\ldots,F$ denote the distinct fortnights in the study and let $n_{f}$ denote the number of observations in fortnight $f$. Let $Z$ denote the complete data (with 11256 observations), consisting of an indicator for seroreactivity ${(Y}_{i})$, the corresponding fortnight of the sample ${(f}_{i})$ and the total number of observations from fortnight $f_{i}$ ($n_{f_{i}})$.

Let $b=1,\ldots,1000$ denote the bootstrapping sample and let $Z^{b}$ denote the data obtained by sampling with replacement $B=11256$ times from $Z$ under the $b$th sample.

Prevalence of seroreactivity per fortnight of the $b$th bootstrapping sample was then calculated as follows:

$$\hat{s}_{b,f}=\frac{\sum_{Y_{b,i}\in f} Y_{b,i}}{n_{b,f}}$$

where $Y_{b,i}$ is the $i$th observation of $Z^{b}$ and $n_{b,f}$ is the number of observations in fortnight $f$ under bootstrap sample $b$.

Denote by $g()$ the shape constrained P-splines in which monotonicity between knots is enforced (as detailed above). This function was applied to $\hat{s}_{b,f}$ in order to obtain smoothed estimates of prevalence of seroreactivity, denoted by $\hat{s}_{b,f,smooth}=g(\hat{s}_{b,f})$.

Let $I_{b,f}$ denote the incidence of the $b$th bootstrapping sample for fortnight $f$. We therefore have that

$I_{b,f}= \frac{\hat{s}_{b,f,smooth}- \hat{s}_{b,f-1,smooth}}{1- \hat{s}_{b,f-1,smooth}}$.

Supplementary Figure 2 presents estimates of the incidence of seroreactivity among susceptible persons.

**Supplementary Figure 2: Estimated incidence of seroreactivity among susceptible persons over time with 95% bootstrapped confidence intervals. The red dotted line indicates the introduction of the first “lockdown” in the UK.**


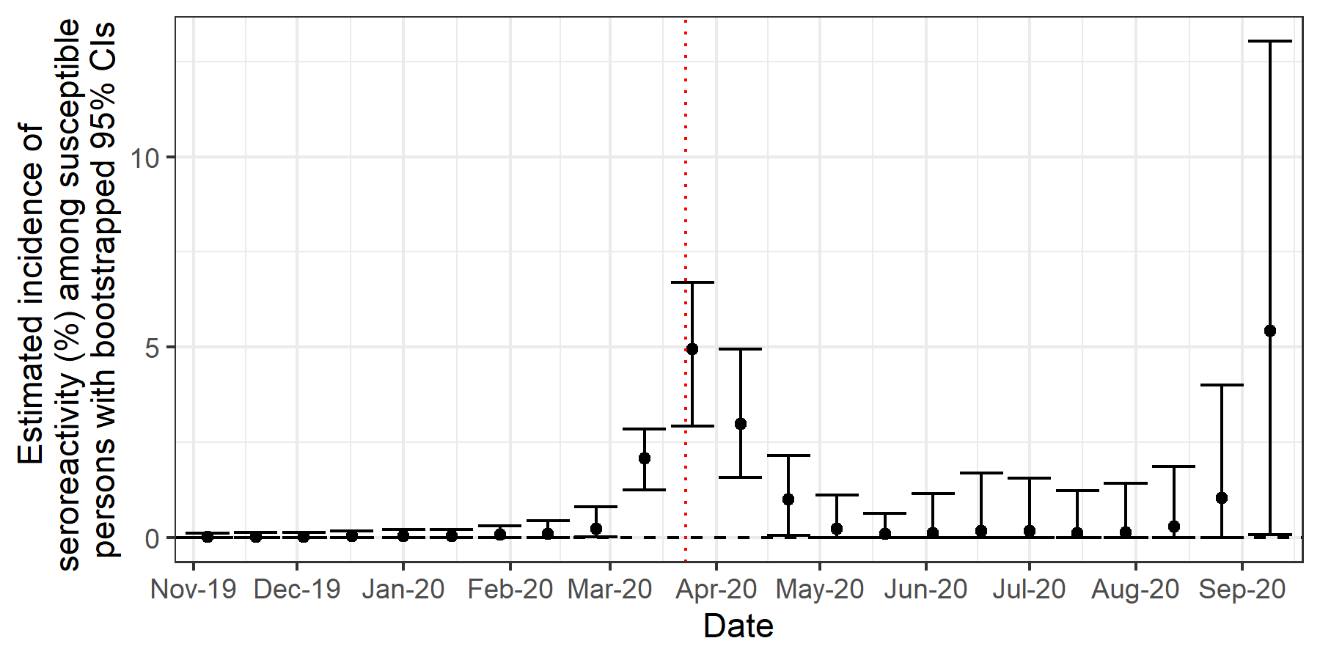


**Comparison of estimates with REACT-2 study**

Supplementary Figure 3 presents our estimates of the prevalence of seroreactivity along with the estimates from the REACT-2 study at a national-level and only within London.^6^

**Supplementary Figure 3: Estimates of prevalence of seroreactivity using historic antenatal samples in this study (black) compared to 18 – 44-year-old women across our ethnicity and IMD groups from REACT-2 at a national-level (dark green) and only within London (light green).**


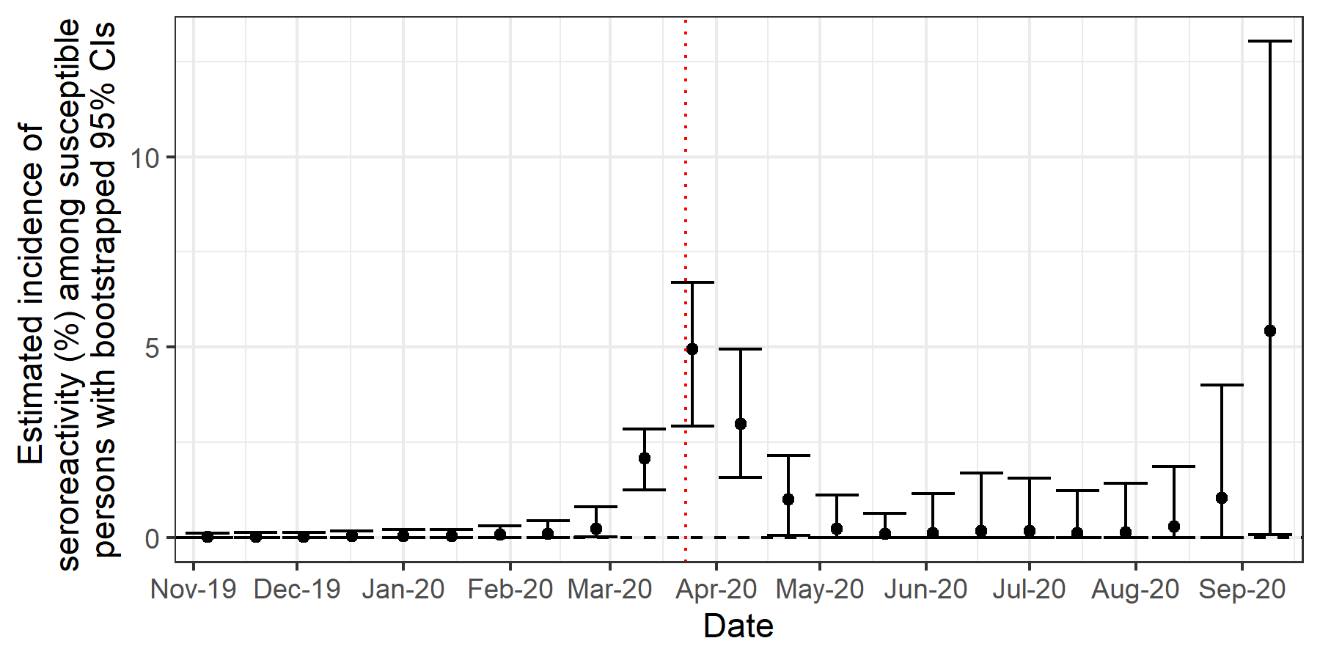


**Internal and External Validation of June 2019 seroreactive and non-seroreactive samples**

Supplementary Table 3 presents validation of DABA testing of June 2019 samples, whereby the 8 seroreactive samples in that period and another 16 consecutive negative controls were re-tested blind at UKHSA Colindale with NP capture and S1 capture assays. No positive samples by DABA were positive for NP or S1 capture. After blocking for seasonal coronaviruses, all non-seroreactive samples were confirmed as non-seroreactive.

Positive samples were also re-tested, unblinded, at Imperial College London on S1 capture assays. One of the eight samples seroreactive on DABA was seroreactive on S1 capture IgM assay (Supplementary Table 3).

**Supplementary Table 3: Binding ratios (BR) of validatory testing conducted on June 2019 samples seroreactive under DABA assay along with 16 consecutive negative controls. Samples were re-tested blind at UKHSA (PHE) with NP capture and S1 capture assays. Positive samples were also re-tested, unblinded, at Imperial College London on S1 capture assays. Under all assays, a sample is considered seroreactive if BR ≥1.**

| **Assay** | **DABA (BR)** | **PHE NP Capture (No block) (BR)** | **PHE NP Capture (with block) (BR)** | **PHE S1 Capture (BR)** | **Imperial S1 Capture Assay for ImmunoglobulinA (BR)** | **Imperial S1 Capture Assay for Immunoglobulin G (BR)** | **Imperial S1 Capture Assay for Immunoglobulin M (BR)** |
| --- | --- | --- | --- | --- | --- | --- | --- |
| **June 2019 samples tested positive under DABA** | 2.20 | 0.17 | 0.20 | 0.13 | 0.10 | 0.20 | 0.40 |
|  | 11.94 | 0.17 | 0.19 | 0.13 | 0.20 | 0.10 | 1.30 |
|  | 2.43 | 0.27 | 0.30 | 0.13 | 0.20 | 0.20 | 0.70 |
|  | 1.98 | 0.17 | 0.21 | 0.13 | 0.20 | 0.20 | 0.40 |
|  | 1.87 | 0.21 | 0.18 | 0.13 | 0.30 | 0.20 | 0.50 |
|  | 2.49 | 0.22 | 0.25 | 0.18 | 0.20 | 0.20 | 0.30 |
|  | 1.81 | 0.18 | 0.16 | 0.13 | 0.20 | 0.20 | 0.20 |
|  | 1.18 | 0.17 | 0.28 | 0.13 | 0.20 | 0.20 | 0.40 |
| **June 2019 samples tested negative on DABA, consecutive controls** | 0.20 | 0.46 | 0.23 | 0.13 |  | | |
|  | 0.30 | 0.17 | 0.19 | 0.12 |  |  |  |
|  | 0.21 | 0.29 | 0.25 | 0.16 |  |  |  |
|  | 0.10 | 0.75 | 0.21 | 0.18 |  |  |  |
|  | 0.23 | 0.19 | 0.30 | 0.13 |  |  |  |
|  | 0.13 | 0.53 | 0.41 | 0.13 |  |  |  |
|  | 0.10 | 0.26 | 0.36 | 0.13 |  |  |  |
|  | 0.15 | 0.19 | 0.19 | 0.13 |  |  |  |
|  | 0.27 | 0.21 | 0.17 | 0.12 |  |  |  |
|  | 0.32 | 0.21 | 0.19 | 0.13 |  |  |  |
|  | 0.21 | 0.21 | 0.25 | 0.18 |  |  |  |
|  | 0.16 | 0.29 | 0.19 | 0.13 |  |  |  |
|  | 0.09 | 1.05 | 0.15 | 0.13 |  |  |  |
|  | 0.60 | 0.20 | 0.16 | 0.13 |  |  |  |
|  | 0.21 | 0.22 | 0.17 | 0.11 |  |  |  |
|  | 0.18 | 0.99 | 0.22 | 0.18 |  |  |  |

**References**

1. Coronavirus (COVID-19) in the UK. *gov.uk* (2022).

2. Buitrago-Garcia, D. *et al.* Occurrence and transmission potential of asymptomatic and presymptomatic SARS-CoV-2 infections: A living systematic review and meta-analysis. *PLoS Med.* **17**, e1003346 (2020).

3. Lessler, J. *et al.* Estimating the Severity and Subclinical Burden of Middle East Respiratory Syndrome Coronavirus Infection in the Kingdom of Saudi Arabia. *Am. J. Epidemiol.* **183**, 657–663 (2016).

4. Weaver, M. Daughter calls for Kent man to be recognised as UK’s first Covid victim. *The Guardian* (2021).

5. Metcalf, C. J. E. *et al.* Use of serological surveys to generate key insights into the changing global landscape of infectious disease. *Lancet* **388**, 728–730 (2016).

6. Real-time Assessment of Community Transmission (REACT) Study. *Imperial College London* (2021).

7. Coronavirus (COVID-19) Infection Survey: England. *Office for National Statistics* (2021).

8. Dickson, E. *et al.* Enhanced surveillance of COVID-19 in Scotland: population-based seroprevalence surveillance for SARS-CoV-2 during the first wave of the epidemic. *Public Health* **190**, 132–134 (2021).

9. Thompson, C. P. *et al.* Detection of neutralising antibodies to SARS-CoV-2 to determine population exposure in Scottish blood donors between March and May 2020. *Euro Surveill. Bull. Eur. sur les Mal. Transm. = Eur. Commun. Dis. Bull.* **25**, (2020).

10. Public Health England. *Sero-surveillance of COVID-19*. (2021).

11. Lumley, S. F. *et al.* SARS-CoV-2 antibody prevalence, titres and neutralising activity in an antenatal cohort, United Kingdom, 14 April to 15 June 2020. *Eurosurveillance* **25**, (2020).

12. Public Health England. *Infectious diseases in pregnancy screening (IDPS): programme overview*. (2021).

13. NHS Digital. *Maternity Services Monthly Statistics June 2019, experimental statistics*. (2019).

14. Public Health England. *Health of women before and during pregnancy: health behaviours, risk factors and inequalities: An updated analysis of the maternity services dataset antenatal booking data*. (2019).

15. Rosadas, C., Randell, P., Khan, M., McClure, M. O. & Tedder, R. S. Testing for responses to the wrong SARS-CoV-2 antigen? *Lancet* **396**, e23 (2020).

16. El Bouzidi, K. *et al.* Severe Acute Respiratory Syndrome Coronavirus-2 Infections in Critical Care Staff: Beware the Risks Beyond the Bedside. *Crit. Care Med.* **49**, 428–436 (2021).

17. Rosadas, C. *et al.* Detection and quantification of antibody to SARS CoV 2 receptor binding domain provides enhanced sensitivity, specificity and utility. *J. Virol. Methods* **302**, 114475 (2022).

18. Riley, S. *et al.* REACT-1 round 9 final report: Continued but slowing decline of prevalence of SARS-CoV-2 during national lockdown in England in February 2021. *medRxiv* (2021).

19. Mahase, E. Coronavirus: UK screens direct flights from Wuhan after US case. *BMJ* **368**, m265 (2020).

20. Razai, M. S., Kankam, H. K. N., Majeed, A., Esmail, A. & Williams, D. R. Mitigating ethnic disparities in covid-19 and beyond. *BMJ* **372**, m4921 (2021).

21. Mathur, R. *et al.* Ethnic differences in SARS-CoV-2 infection and COVID-19-related hospitalisation, intensive care unit admission, and death in 17 million adults in England: an observational cohort study using the OpenSAFELY platform. *Lancet* **397**, 1711–1724 (2021).

22. Ward, H. *et al.* SARS-CoV-2 antibody prevalence in England following the first peak of the pandemic. *Nat. Commun.* **12**, 905 (2021).

23. Ministry of Housing Communities & Local Government. *English Indices of Deprivation*. (2019).

24. Fraley, E. *et al.* Cross-reactive antibody immunity against SARS-CoV-2 in children and adults. *Cell. Mol. Immunol.* **18**, 1826–1828 (2021).

25. Bates, T. A. *et al.* Cross-reactivity of SARS-CoV structural protein antibodies against SARS-CoV-2. *Cell Rep.* **34**, 108737 (2021).

26. Rogan, W. J. & Gladen, B. Estimating prevalence from the results of a screening test. *Am. J. Epidemiol.* **107**, 71–76 (1978).

27. Apolone, G. *et al.* Unexpected detection of SARS-CoV-2 antibodies in the prepandemic period in Italy. *Tumori J.* 0300891620974755 (2020) doi:10.1177/0300891620974755.

28. Parodi, E. WHO asks for re-checks of research on when coronavirus first surfaced in Italy. *Reuters* (2021).

29. Kontopantelis, E., Mamas, M. A., Deanfield, J., Asaria, M. & Doran, T. Excess mortality in England and Wales during the first wave of the COVID-19 pandemic. *J. Epidemiol. Community Health* **75**, 213 LP – 223 (2021).

30. National Pathology Accreditation Advisory Council. *Requirements for the retention of laboratory records and diagnostic material (Seventh Edition 2018)*. (2018).

31. Jamieson, D. J. & Rasmussen, S. A. An update on COVID-19 and pregnancy. *Am. J. Obstet. Gynecol.* **226**, 177–186 (2022).

32. Tapper, J. Dismay as funding for UK’s ‘world-beating’ Covid trackers is axed. *The Observer* (2022).

33. Daly, J. COVID sewage surveillance scaled back, but experts say data is crucial. *ABC News* (2022).
