# Supplementary material for: Tracking the incidence and risk factors for SARS-CoV-2 infection using historical maternal booking serum samples
Source: PLoS One. 2022 Sep 2;17(9):e0273966. doi: 10.1371/journal.pone.0273966 (PMC9439206; doi:10.1371/journal.pone.0273966)
Supplement: Attachment — Submitted filename: tracking the incidence of COVID-19 reviers comment.docx [file pone.0273966.s002.docx]

Title: Tracking the incidence and risk factors for SARS-CoV-2 infection using historical maternal booking serum samples

As a scientific procedure your paper needs minor revision for further improvement. So please kindly consider these issues raised by the reviewers and the editor.

Editor comment:

1. Please abide by the journal guideline
2. Check the grammar
3. Data availability is mandatory so please attach the data

Reviewer 1

In this manuscript, Mullins and McCabe et al. have estimated the prevalence of seroreactivity of total antibodies to the severe acute respiratory syndrome coronavirus 2 (SARS-CoV-2) receptor-binding domain (anti-RBD) in stored antenatal serum samples over time between COVID-19 pre-pandemic and pandemic periods. The authors reported a higher prevalence of seroreactivity to SARS-CoV-2 on Sept 2019 compared to mid-Feb 2020. Overall it is an interesting study representing a retrospective analysis of the prevalence of seroreactivity over time and its association with variables. My comments to the authors are:

1. In the method, in the logistic regression, have you controlled potential confounders (such as BMI, previous history of flu infections, flu vaccination status etc.) potentially affected the outcome variable in the analysis?
2. In the method ‘Defining seroreactivity’ section, please provide rationale and/or reference why the binding ratio (BR) ≥ 1 was considered as seroreactive for SARS-CoV-2 anti-RBD.
3. In the result, table 3, is it adjusted or unadjusted odds?
4. Some minor errors in the use of English throughout, i.e, missing/incorrect use of articles and prepositions - further proofreading required.

Reviewer 2

First of all, I would like to thank all authors.... this manuscript titled “Tracking the incidence and risk factors for SARS-CoV-2 infection using historical maternal booking serum samples" is easy to follow and well written. However, I have only one comment:
Could you please follow PLOS ONE guidelines, such as line number and reference style?

Reviewer 3 comment

1. Would like to hear more about the choice of sample size prior to starting the study. Maybe mention the possible limitations of generalizability of maternal cohorts when pregnant women may be more susceptible to infection.
