# Supplementary material for: Correction: Tracking the incidence and risk factors for SARS-CoV-2 infection using historical maternal booking serum samples
Source: PLoS One. 2024 Aug 1;19(8):e0308512. doi: 10.1371/journal.pone.0308512 (PMC11293627; doi:10.1371/journal.pone.0308512)
Supplement: S1 File — (PDF) [file pone.0308512.s001.pdf]

Tracking the incidence and risk factors for SARS-CoV-2 infection using historical maternal booking serum samples

Supplementary Material

Edward W. S. Mullins*¶, Ruth McCabe¶, Sheila M. Bird, Paul Randell, Marcus J. Pond, Lesley Regan, Eleanor Parker, Myra McClure, Christl A. Donnelly

¶ These authors contributed equally to this work.

*Corresponding author

Email: edward.mullins@imperial.ac.uk.

Sample size

Supplementary Table 1 presents an overview of the number of samples categorised as 'clearly' and 'borderline' seroreactive or non-seroreactive.

Supplementary Table 1: Classification and sample sizes of observations from October 2019 to September 2020 as seroreactive and non-seroreactive according to different thresholds of binding ratio (BR) values.

Assay threshold binding ratios (BRs)	Interpretation	Threshold sample size	Cumulative sample size
BR ≥ 1.2	Clearly seroreactive	665	665
1.2 > BR ≥ 1	Borderline seroreactive	35	700
1 > BR ≥ 0.8	Borderline non-seroreactive	70	770
0.8 > BR	Clearly non-seroreactive	10,486	11,256

Estimating prevalence of seroreactivity

Let n be the total number of observations. For each observation i, Y_i denotes whether this observation is seroreactive or non-seroreactive:

$$Y_i = \begin{cases} 1 \; if \; observation \; is \; seroreactive \\ 0 \; if \; observation \; is \; non-seroreactive \end{cases}$$

Each observation can be considered an independent Bernoulli trial for seroreactivity with probability s, where s denotes prevalence of seroreactivitiy. It follows that the total number of seroreactive observations, $X = x$, is a binomially-distributed random variable with parameters n and s:

$$X = \sum_{i=1}^{n} Y_i \sim Binomial(n, s)$$

Therefore, prevalence of seroreactivity was naively estimated using the maximum likelihood estimator (MLE) of the binomial distribution as follows:

$$\hat{s} = \frac{x}{n}$$

where the number of reactive observations was measured according to clearly and borderline seroreactive observations (binding ratio (BR) value ≥ 1). 95% confidence intervals were generated using the exact binomial method by solving the following equations for s_L and s_U, respectively:

$$\sum_{k=0}^{x} \binom{n}{k} s_U^k (1 - s_U)^{n-k} = \frac{\alpha}{2}$$

$$\sum_{k=0}^{x-1} \binom{n}{k} s_L^k (1 - s_L)^{n-k} = 1 - \frac{\alpha}{2}$$

where $\alpha = 0.05$.

Fisher's exact test for prevalence in June 2019 compared to main study period

Supplementary Table 2 presents the results of the two-sided Fisher's exact tests to test for the significance in the odds of a seroreactive sample between June 2019 compared to each fortnight in the main study.

Supplementary Table 2: Results of two-sided Fisher's exact test in the odds of observing a seroreactive sample in June 2019 (1000 samples, 8 positives) in comparison to each fortnight in the main study period (October 2019 – September 2020).

Collection fortnight (fortnight-year)	Calendar date	Total observations (n)	Seroreactive observations (x)	Odds Ratio (MLE (95% exact confidence intervals))	p-value
22-2019	22/10/2019 - 04/11/2019	608	8	0.61 (0.20 – 1.86)	0.313
23-2019	05/11/2019 - 18/11/2019	610	5	0.98 (0.28 – 3.81)	1.000
24-2019	19/11/2019 - 02/12/2019	546	6	0.73 (0.22 – 2.55)	0.581
25-2019	03/12/2019 - 16/12/2019	514	3	1.38 (0.33 – 8.07)	0.759
26-2019	17/12/2019 - 31/12/2019	554	5	0.89 (0.25 – 3.46)	0.781
1-2020	01/01/2020 - 14/01/2020	467	5	0.75 (0.21 – 2.91)	0.565
2-2020	15/01/2020 - 28/01/2020	572	6	0.76 (0.23 – 2.67)	0.590
3-2020	29/01/2020 - 11/02/2020	669	7	0.76 (0.24 – 2.48)	0.607
4-2020	12/02/2020 - 25/02/2020	530	9	1.27 (0.47 – 0.16)	0.127
5-2020	26/02/2020 - 10/03/2020	628	7	0.72 (0.23 – 2.33)	0.597
6-2020	11/03/2020 - 24/03/2020	659	18	0.29 (0.11 – 0.70)	0.004
7-2020	25/03/2020 - 07/04/2020	392	34	0.09 (0.03 – 0.19)	<0.001
8-2020	08/04/2020 - 21/04/2020	622	73	0.06 (0.03 – 0.13)	<0.001
9-2020	22/04/2020 - 05/05/2020	637	77	0.06 (0.02 – 0.12)	<0.001
10-2020	06/05/2020 - 19/05/2020	584	83	0.05 (0.02 – 0.10)	<0.001
11-2020	20/05/2020 - 02/06/2020	482	53	0.07 (0.03 – 0.14)	<0.001
12-2020	03/06/2020 - 16/06/2020	499	58	0.06 (0.03 – 0.13)	<0.001
13-2020	17/06/2020 - 30/06/2020	278	38	0.05 (0.02 – 0.11)	<0.001
14-2020	01/07/2020 - 14/07/2020	254	37	0.05 (0.02 – 0.11)	<0.001
15-2020	15/07/2020 - 28/07/2020	288	35	0.06 (0.02 – 0.13)	<0.001
16-2020	29/07/2020 - 11/08/2020	267	40	0.05 (0.02 – 0.10)	<0.001
17-2020	12/08/2020 - 25/08/2020	198	25	0.06 (0.02 – 0.13)	<0.001
18-2020	26/08/2020 - 08/09/2020	233	35	0.05 (0.02 – 0.10)	<0.001
19-2020	09/09/2020 - 22/09/2020	165	33	0.03 (0.01 – 0.07)	<0.001

Logistic regression

The logistic regression model was formally described as:

$$
\begin{aligned}
logit(s) = \beta_0 &+ \beta_1 Age_{18-29} + \beta_2 Age_{30-34} \\
&+ \beta_3 Ethnicity_{all-black} + \beta_4 Ethnicity_{all-Asian} + \beta_5 Ethnicity_{other} \\
&+ \beta_6 IMD_{deciles\ 3-6} + \beta_7 IMD_{deciles\ 7-10} \\
&+ \beta_8 Fortnight_{2019-22} + \beta_9 Fortnight_{2019-23} + \beta_{10} Fortnight_{2019-24} \\
&+ \beta_{11} Fortnight_{2019-25} + \beta_{12} Fortnight_{2019-26} + \beta_{13} Fortnight_{2020-1} \\
&+ \beta_{14} Fortnight_{2020-2} + \beta_{15} Fortnight_{2020-3} + \beta_{16} Fortnight_{2020-4} \\
&+ \beta_{17} Fortnight_{2020-5} + \beta_{18} Fortnight_{2020-7} + \beta_{19} Fortnight_{2020-8} \\
&+ \beta_{20} Fortnight_{2020-9} + \beta_{21} Fortnight_{2020-10} + \beta_{22} Fortnight_{2020-11} \\
&+ \beta_{23} Fortnight_{2020-12} + \beta_{24} Fortnight_{2020-13} + \beta_{25} Fortnight_{2020-14} \\
&+ \beta_{26} Fortnight_{2020-15} + \beta_{27} Fortnight_{2020-16} + \beta_{28} Fortnight_{2020-17} \\
&+ \beta_{29} Fortnight_{2020-18} + \beta_{30} Fortnight_{2020-19}
\end{aligned}
$$

The addition model with the interaction term between ethnicity and IMD decile was as follows:

$$
\begin{aligned}
logit(s) = \beta_0 &+ \beta_1 Age_{18-29} + \beta_2 Age_{30-34} \\
&+ \beta_3 Ethnicity_{all-black} + \beta_4 Ethnicity_{all-Asian} + \beta_5 Ethnicity_{other} \\
&+ \beta_6 IMD_{deciles\ 3-6} + \beta_7 IMD_{deciles\ 7-10} \\
&+ \beta_8 Fortnight_{2019-22} + \beta_9 Fortnight_{2019-23} + \beta_{10} Fortnight_{2019-24} \\
&+ \beta_{11} Fortnight_{2019-25} + \beta_{12} Fortnight_{2019-26} + \beta_{13} Fortnight_{2020-1} \\
&+ \beta_{14} Fortnight_{2020-2} + \beta_{15} Fortnight_{2020-3} + \beta_{16} Fortnight_{2020-4} \\
&+ \beta_{17} Fortnight_{2020-5} + \beta_{18} Fortnight_{2020-7} + \beta_{19} Fortnight_{2020-8} \\
&+ \beta_{20} Fortnight_{2020-9} + \beta_{21} Fortnight_{2020-10} + \beta_{22} Fortnight_{2020-11} \\
&+ \beta_{23} Fortnight_{2020-12} + \beta_{24} Fortnight_{2020-13} + \beta_{25} Fortnight_{2020-14} \\
&+ \beta_{26} Fortnight_{2020-15} + \beta_{27} Fortnight_{2020-16} + \beta_{28} Fortnight_{2020-17} \\
&+ \beta_{29} Fortnight_{2020-18} + \beta_{30} Fortnight_{2020-19} \\
&+ \beta_{31} Ethnicity_{all-black} \times IMD_{deciles\ 3-6} \\
&+ \beta_{32} Ethnicity_{all-Asian} \times IMD_{deciles\ 3-6} \\
&+ \beta_{33} Ethnicity_{other} \times IMD_{deciles\ 3-6} \\
&+ \beta_{34} Ethnicity_{all-black} \times IMD_{deciles\ 7-10} \\
&+ \beta_{35} Ethnicity_{all-Asian} \times IMD_{deciles\ 7-10} \\
&+ \beta_{36} Ethnicity_{other} \times IMD_{deciles\ 7\ -10}
\end{aligned}
$$

Baseline fortnight selection

Fortnight 6 was just before the first UK lockdown in March 2020, was likely to coincide with high rates of infection and was selected as the baseline fortnight.

Incidence of seroreactivity

Supplementary Figure 1 presents the shape constrained P-spline fitted to the estimates of prevalence of seroreactivity over time, in which monotonicity between knots was enforced. In this instance, we specified 24 knots, corresponding to the number of fortnights for which incidence was estimated. The smoothed curve provided the estimates from which incidence was derived.

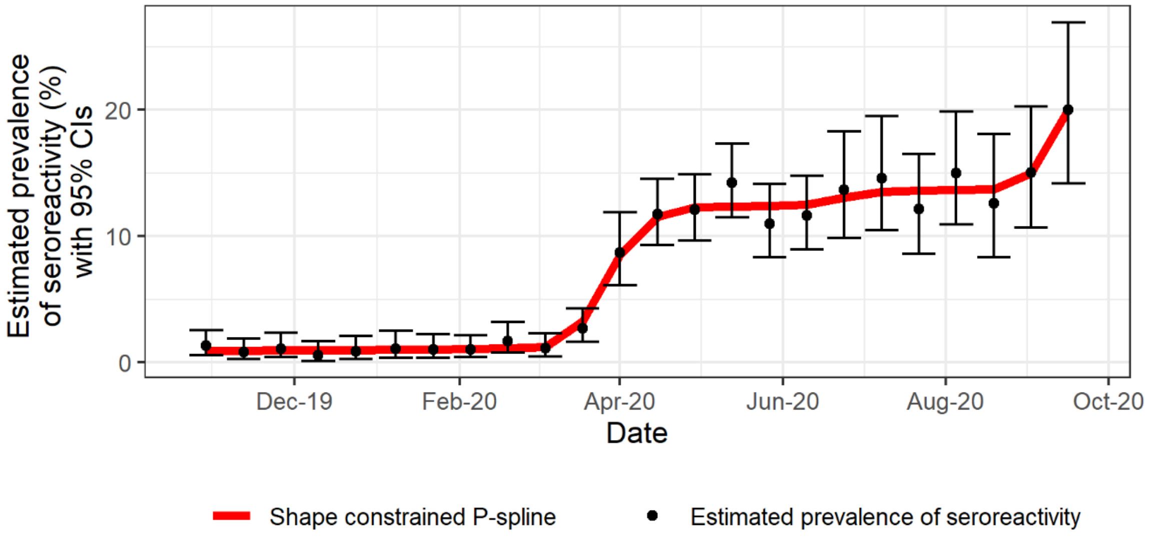

Supplementary Figure 1: Shape constrained P-spline (red) fit to prevalence of seroreactivity estimated using entire cleaned data set (black).

The bootstrapping procedure is presented below.

Let $f = 1, \ldots, F$ denote the distinct fortnights in the study and let n_f denote the number of observations in fortnight f. Let Z denote the complete data (with 11256 observations), consisting of an indicator for seroreactivity (Y_i), the corresponding fortnight of the sample (f_i) and the total number of observations from fortnight f_i (n_{f_i}).

Let $b = 1, \ldots, 1000$ denote the bootstrapping sample and let Z^b denote the data obtained by sampling with replacement $B = 11256$ times from Z under the bth sample.

Prevalence of seroreactivity per fortnight of the bth bootstrapping sample was then calculated as follows:

$$\hat{s}_{b,f} = \frac{\sum_{Y_{b,i} \in f} Y_{b,i}}{n_{b,f}}$$

where $Y_{b,i}$ is the ith observation of Z^b and $n_{b,f}$ is the number of observations in fortnight f under bootstrap sample b.

Denote by $g()$ the shape constrained P-splines in which monotonicity between knots is enforced (as detailed above). This function was applied to $\hat{s}_{b,f}$ in order to obtain smoothed estimates of prevalence of seroreactivity, denoted by $\hat{s}_{b,f,smooth} = g(\hat{s}_{b,f})$.

Let $I_{b,f}$ denote the incidence of the bth bootstrapping sample for fortnight f. We therefore have that

$$I_{b,f} = \frac{\hat{s}_{b,f,smooth} - \hat{s}_{b,f-1,smooth}}{1 - \hat{s}_{b,f-1,smooth}}.$$

Supplementary Figure 2 presents estimates of the incidence of seroreactivity among susceptible persons.

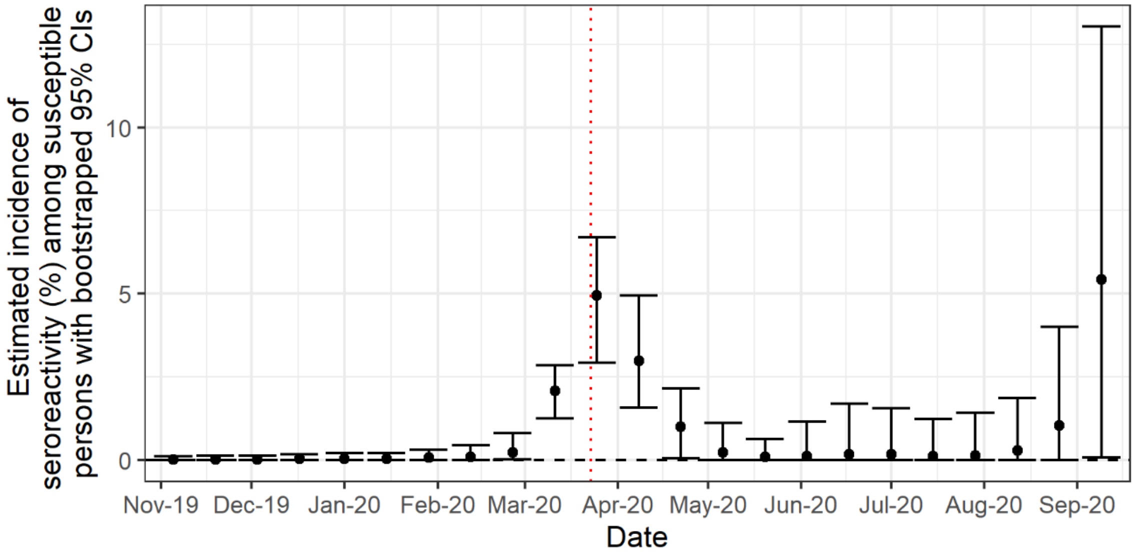

Supplementary Figure 2: Estimated incidence of seroreactivity among susceptible persons over time with 95% bootstrapped confidence intervals. The red dotted line indicates the introduction of the first "lockdown" in the UK.

Comparison of estimates with REACT-2 study

Supplementary Figure 3 presents our estimates of the prevalence of seroreactivity along with the estimates from the REACT-2 study at a national-level and only within London.[1]

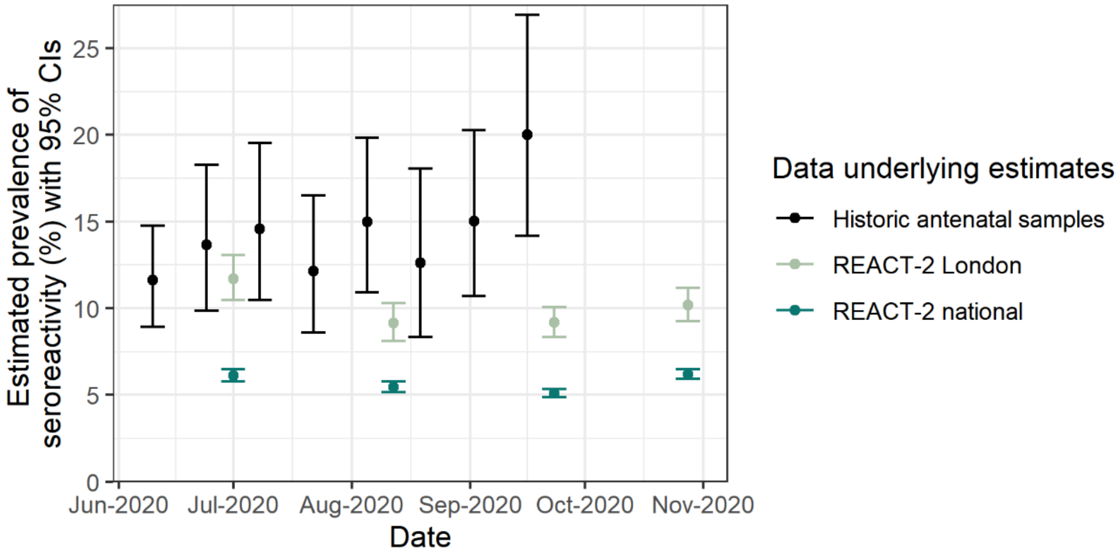

Supplementary Figure 3: Estimates of prevalence of seroreactivity using historic antenatal samples in this study (black) compared to 18 – 44-year-old women across our ethnicity and IMD groups from REACT-2 at a national-level (dark green) and only within London (light green).

Internal and External Validation of June 2019 seroreactive and non-seroreactive samples

Supplementary Table 3 presents validation of DABA testing of June 2019 samples, whereby the 8 seroreactive samples in that period and another 16 consecutive negative controls were re-tested blind at UKHSA Colindale with NP capture and S1 capture assays. No positive samples by DABA were positive for NP or S1 capture. After blocking for seasonal coronaviruses, all non-seroreactive samples were confirmed as non-seroreactive.

Positive samples were also re-tested, unblinded, at Imperial College London on S1 capture assays. One of the eight samples seroreactive on DABA was seroreactive on S1 capture IgM assay (Supplementary Table 3).

Supplementary Table 3: Binding ratios (BR) of validatory testing conducted on June 2019 samples seroreactive under DABA assay along with 16 consecutive negative controls. Samples were re-tested blind at UKHSA (PHE) with NP capture and S1 capture assays. Positive samples were also re-tested, unblinded, at Imperial College London on S1 capture assays. Under all assays, a sample is considered seroreactive if BR ≥1.

Assay	DABA (BR)	PHE NP Capture (No block) (BR)	PHE NP Capture (with block) (BR)	PHE S1 Capture (BR)	Imperial S1 Capture Assay for ImmunoglobulinA (BR)	Imperial S1 Capture Assay for Immunoglobulin G (BR)	Imperial S1 Capture Assay for Immunoglobulin M (BR)
June 2019 samples tested positive under DABA	2.20	0.17	0.20	0.13	0.10	0.20	0.40
	11.94	0.17	0.19	0.13	0.20	0.10	1.30
	2.43	0.27	0.30	0.13	0.20	0.20	0.70
	1.98	0.17	0.21	0.13	0.20	0.20	0.40
	1.87	0.21	0.18	0.13	0.30	0.20	0.50
	2.49	0.22	0.25	0.18	0.20	0.20	0.30
	1.81	0.18	0.16	0.13	0.20	0.20	0.20
	1.18	0.17	0.28	0.13	0.20	0.20	0.40
June 2019 samples tested negative on DABA, consecutive controls	0.20	0.46	0.23	0.13			
	0.30	0.17	0.19	0.12			
	0.21	0.29	0.25	0.16			
	0.10	0.75	0.21	0.18			
	0.23	0.19	0.30	0.13			
	0.13	0.53	0.41	0.13			
	0.10	0.26	0.36	0.13			
	0.15	0.19	0.19	0.13			
	0.27	0.21	0.17	0.12			
	0.32	0.21	0.19	0.13			
	0.21	0.21	0.25	0.18			
	0.16	0.29	0.19	0.13			
	0.09	1.05	0.15	0.13			
	0.60	0.20	0.16	0.13			
	0.21	0.22	0.17	0.11			
	0.18	0.99	0.22	0.18			

References

1. Real-time Assessment of Community Transmission (REACT) Study. *Imperial College London* https://www.imperial.ac.uk/medicine/research-and-impact/groups/reactstudy/ (2021).
